# Towards non-invasive computational-mechanics and imaging-based diagnostic framework for personalized cardiology for coarctation

**DOI:** 10.1038/s41598-020-65576-y

**Published:** 2020-06-03

**Authors:** Reza Sadeghi, Seyedvahid Khodaei, Javier Ganame, Zahra Keshavarz-Motamed

**Affiliations:** 1 Department of Mechanical Engineering, McMaster University, Hamilton, ON Canada; 2 Division of Cardiology, Department of Medicine, McMaster University, Hamilton, ON Canada; 3 St. Joseph’s Healthcare and Hamilton Health Sciences, Hamilton, ON Canada; 4 School of Biomedical Engineering, McMaster University, Hamilton, ON Canada; 5 School of Computational Science and Engineering, McMaster University, Hamilton, ON Canada

**Keywords:** Biomedical engineering, Mechanical engineering

## Abstract

Coarctation of the aorta (COA) is a congenital narrowing of the proximal descending aorta. Although accurate and early diagnosis of COA hinges on blood flow quantification, proper diagnostic methods for COA are still lacking because fluid-dynamics methods that can be used for accurate flow quantification are not well developed yet. Most importantly, COA and the heart interact with each other and because the heart resides in a complex vascular network that imposes boundary conditions on its function, accurate diagnosis relies on quantifications of the global hemodynamics (heart-function metrics) as well as the local hemodynamics (detailed information of the blood flow dynamics in COA). In this study, to enable the development of new non-invasive methods that can quantify local and global hemodynamics for COA diagnosis, we developed an innovative fast computational-mechanics and imaging-based framework that uses Lattice Boltzmann method and lumped-parameter modeling that only need routine non-invasive clinical patient data. We used clinical data of patients with COA to validate the proposed framework and to demonstrate its abilities to provide new diagnostic analyses not possible with conventional diagnostic methods. We validated this framework against clinical cardiac catheterization data, calculations using the conventional finite-volume method and clinical Doppler echocardiographic measurements. The diagnostic information, that the framework can provide, is vitally needed to improve clinical outcomes, to assess patient risk and to plan treatment.

## Introduction

Coarctation of the aorta (COA) is a congenital narrowing of the proximal descending aorta. The hemodynamic severity and clinical manifestations of COA vary from asymptomatic mild narrowing of the aortic isthmus to severe obstruction associated with cardiac defects, congestive heart failure and shock in the neonatal period, persistent hypertension and aortic dissection^1^. Not all patients are symptomatic but with disease progression in severity, 60% of adults over 40 with uncorrected COA develop heart failure and 75% of them die by the age of 50, and 90% of them die by the age of 60^2^. Indeed, despite advancements in interventional/surgical techniques, the long-term morbidity and subsequent mortality of patients with COA remain high in comparison with the general population^3,4^.

“Cardiology is flow”^5^ and therefore the essential sources of COA morbidity can be explained on the basis of adverse hemodynamics: abnormal biomechanical forces, abnormal flow patterns - that often characterized by disturbed and turbulent flow- and in some cases by an increase in the heart workload that leads to the development and progression of cardiovascular diseases^5–8^. Flow quantification can be greatly useful for accurate and early diagnosis, but we still lack proper diagnostic methods for many cardiovascular diseases^6,9^, including COA, because the fluid-dynamics methods that can be used as engines of new diagnostic tools are not well developed yet. In this research we contributed to advancing computational mechanics as a powerful means to augment clinical measurements and medical imaging to create novel diagnostic methods for COA at no risk to the patient^6,7^.

The heart resides in a sophisticated vascular network whose loads impose boundary conditions on the heart function^6,7,10–12^. Effective diagnosis of COA hinges on: (1) quantifications of the global hemodynamics (heart function metrics, e.g., left ventricle workload and instantaneous pressure), and (2) quantifications of the local hemodynamics (detailed information of the 3-D flow dynamics in COA). However, there is no method to invasively or noninvasively quantify the heart workload *(global hemodynamics)* while providing contribution breakdown of each component of the cardiovascular system. Moreover, current diagnostic methods cannot quantify details of the flow dynamics of the circulatory system *(local hemodynamics)*. Although all of these can provide valuable information about the patient’s state of cardiac deterioration and heart recovery, currently, clinical decisions are largely made based on the anatomy using medical imaging^9^.

A clinically-useful computational diagnostic framework that can quantify both *local* and *global* hemodynamics for patients with coarctation should satisfy the following 3 requirements:

1. The local fluid dynamics is influenced by the conditions downstream and upstream of coarctation. Therefore, in addition to performing the 3-D blood flow calculations in the patient-specific geometry, imposing accurate patient-specific flow and pressure boundary conditions is critically important for a computational diagnostic framework. This not only gives patient-specific flow and pressure conditions to the local flow but also enables providing diagnostic information about the global circulatory physiology. The patient-specific boundary conditions should be obtained *non-invasively* in each patient because obtaining them invasively (e.g., with catheterization) contradicts the whole purpose of the computational framework.
2. To reliably augment the current clinical diagnostics capabilities with calculations of blood flow through COA, the computational diagnostic framework should be fast enough to provide results in a matter of minutes rather than days.
3. The computational framework should provide valid results to be considered as a reliable diagnostic tool. Upon development of a computational diagnostic framework, its results should be validated against clinical data that include data obtained using cardiac catheterization, Doppler echocardiography and magnetic resonance imaging. Cardiac catheterization is used as the clinical gold standard to evaluate pressure and flow through heart and circulatory system, but it can only provide access to the blood flow and pressure in very limited regions. Doppler echocardiography is the most versatile tool to evaluate local hemodynamics and has a high temporal resolution, but it has limited spatial access through chest. Phase-contrast magnetic resonance imaging can provide local flow, but it is not possible for many patients with implanted devices. As each of these modalities have their own limitations, a multi-modality validation of the computational framework would be required.

There have been attempts for quantifying blood flow through COA (local hemodynamics) using conventional macroscopic numerical methods based on the discretization of Navier–Stokes equations (finite difference method, finite volume method, finite element method, etc.)^11,13–20^. None of these models can satisfy Requirement #2 above because the conventional methods need days of calculations and therefore, they are not feasible for clinical diagnosis. Furthermore, many of these models were restricted to low Reynolds numbers. None of these models satisfy Requirement #3: most were not validated while some were only partially validated. Most of these studies do not satisfy Requirement #1 as they do not have patient-specific boundary conditions. Among all, three studies^14,15,20^ coupled blood-flow calculations with lumped-parameter modelling to impose boundary conditions on the calculations. However, the lumped-parameter models either were not patient specific or needed information from blood-flow measurements using MRI that is not available in all clinics and is not feasible in patients with implanted devices.

Recently, Lattice Boltzmann method (LBM), rooted in mesoscopic kinetic equations^21^, has been developed as a powerful and fast technique for accurate simulations of fluid flow. Since the birth of LBM, there has been an increasing popularity of this method as an alternative to computationally intensive conventional methods for fluid dynamics simulations^22^ because of its simplicity, handling of complex flow phenomena, efficient executions^23^ and the fact that LBM equations can be solved locally and explicitly, and they are intrinsically parallelizable^24^. These promising features have motivated researches to use LBM as the method of choice for computational cardiology^25–28^. Few studies used LBM for the investigation of local hemodynamics of COA without considering any global effects. Although these studies showed effectiveness of LBM for flow analysis, their aim was not developing a diagnosis tool, so they did not satisfy requirements #1 and #3 above^25,29,30^.

In this paper, using LBM and lumped parameter modeling (LPM), we developed an innovative fast computational-mechanics and imaging-based framework that can eventually, upon further development and validation, work as the main component of new diagnostic methods for COA. This computationally fast framework enables (1) quantifying details of 3-D fluid dynamics through the aorta and COA (*local hemodynamics*); (2) quantifying heart function metrics, e.g., left ventricle (LV) workload and instantaneous LV pressure (*global hemodynamics*). Currently, none of the above metrics can be obtained noninvasively in patients and when invasive procedures are undertaken, the collected metrics cannot be as complete as the results that the proposed framework can provide. Our LPM uses a limited number of input parameters all of which can be reliably measured using Doppler echocardiography and a sphygmomanometer with no risks to the patient and thus will make effective and personalized diagnosis possible. Note that the proposed method does not need any catheter data as input parameters of the model. We used clinical data of 3 patients with COA in both pre and post intervention states not only to validate the proposed framework but also to demonstrate its diagnostic abilities by providing novel analyses and interpretations of clinical data. The validation was done against clinical cardiac catheterization data, calculations using the conventional finite-volume method and clinical Doppler echocardiographic measurements. To the best of our knowledge, this is the first study that couple LBM and LPM and satisfies all 3 requirements for developing a clinically-effective computational diagnostic framework to quantify both local and global hemodynamics in patients with COA in both pre and post intervention states.

## Methods

**Figure 1:**
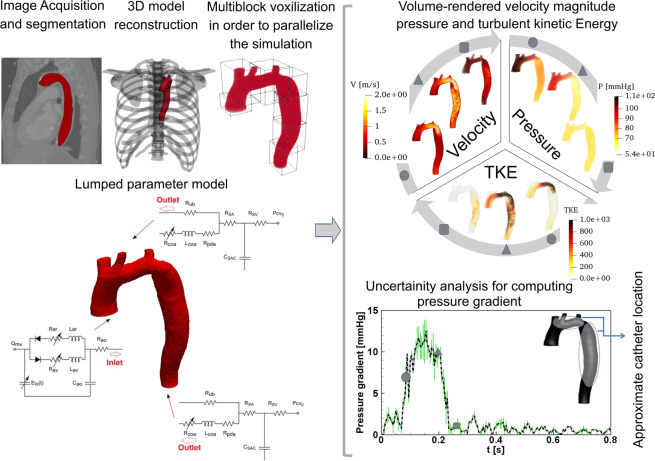
*Reconstructed geometry and simulation domain*. We used CT images from patients to segment and reconstruct the 3D geometries of the complete aorta. These 3-D geometries were used for investigating hemodynamic using computational fluid dynamics. Local flow dynamics is greatly influenced by upstream and downstream flow conditions that are absent in the flow simulation domain. The lumped-parameter model simulates the function of the left side of the heart. Time-dependent inlet flow (at ascending aorta) and outlet pressure (at descending aorta) position were obtained from lumped parameter modeling and applied as the transient boundary conditions. Boundary conditions of the aortic branches were adjusted to match the flow distribution.

**Figure 2:**
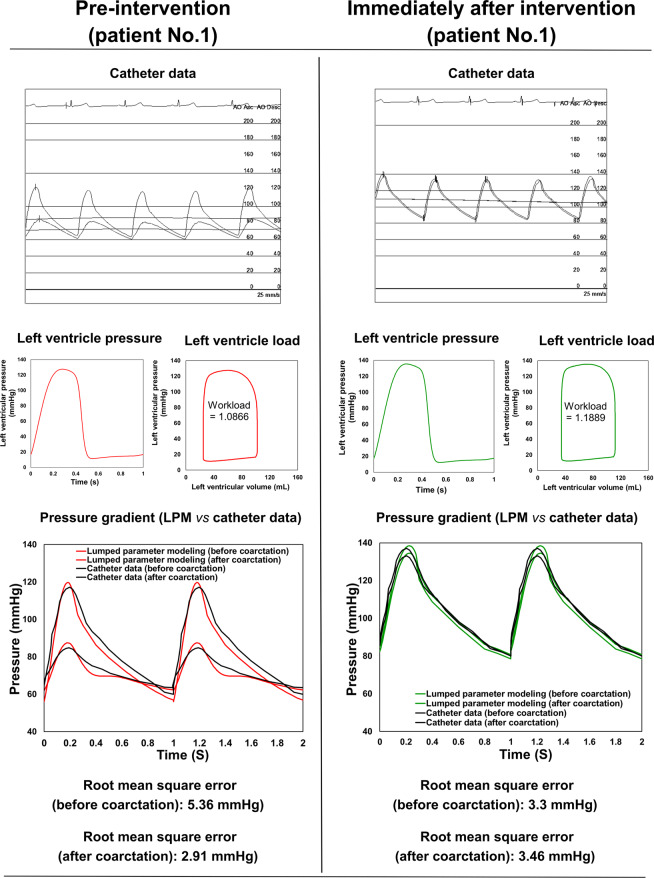
*Validation against catheter data*. Catheter data and results of lumped parameter modeling (aorta and LV pressures and workloads) in Patient No. 1.

**Figure 3:**
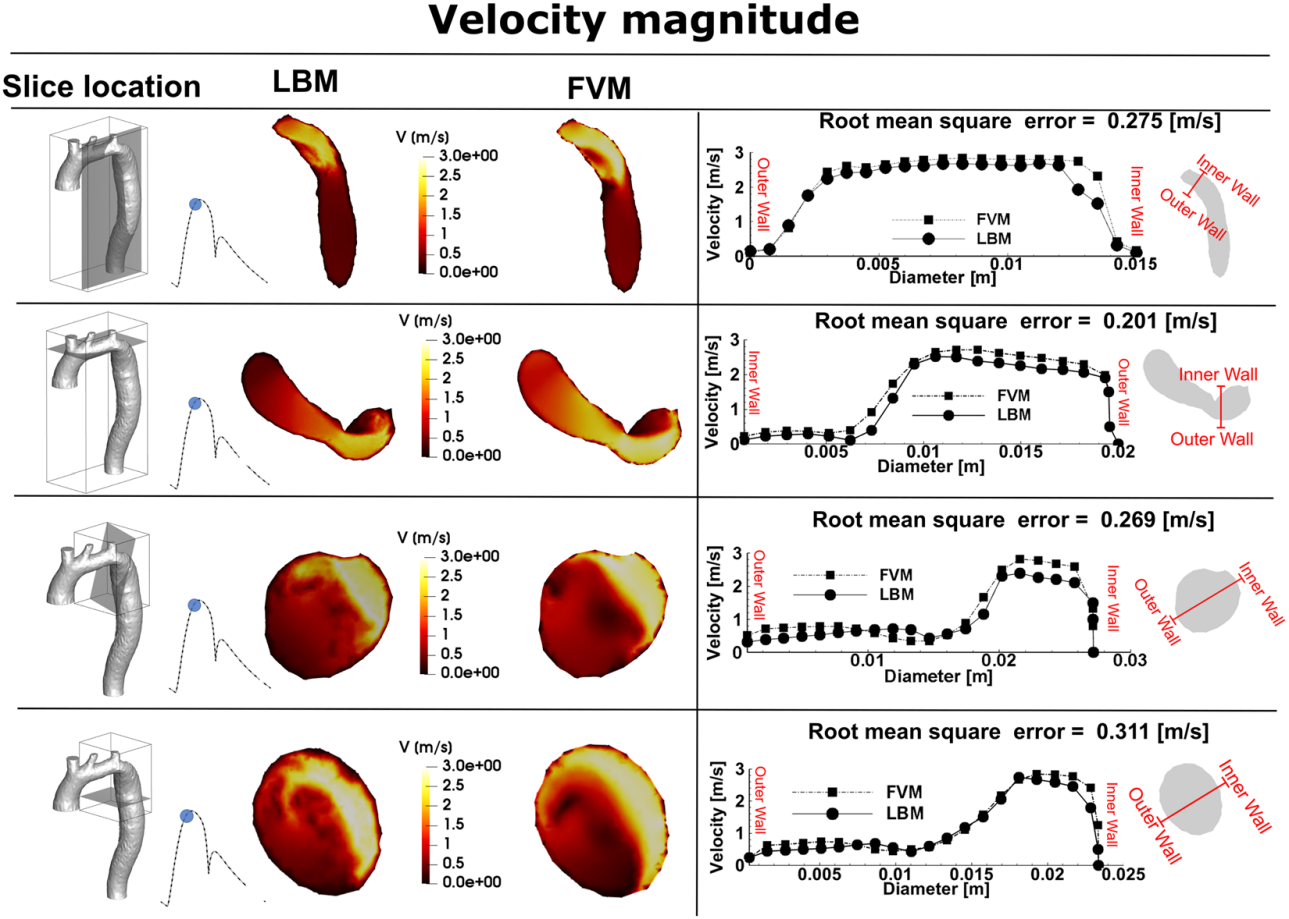
*Velocity comparison*. Velocity at different cross sections of the aorta, simulated using lattice Boltzmann method (LBM) and finite volume method (FVM).

**Figure 4:**
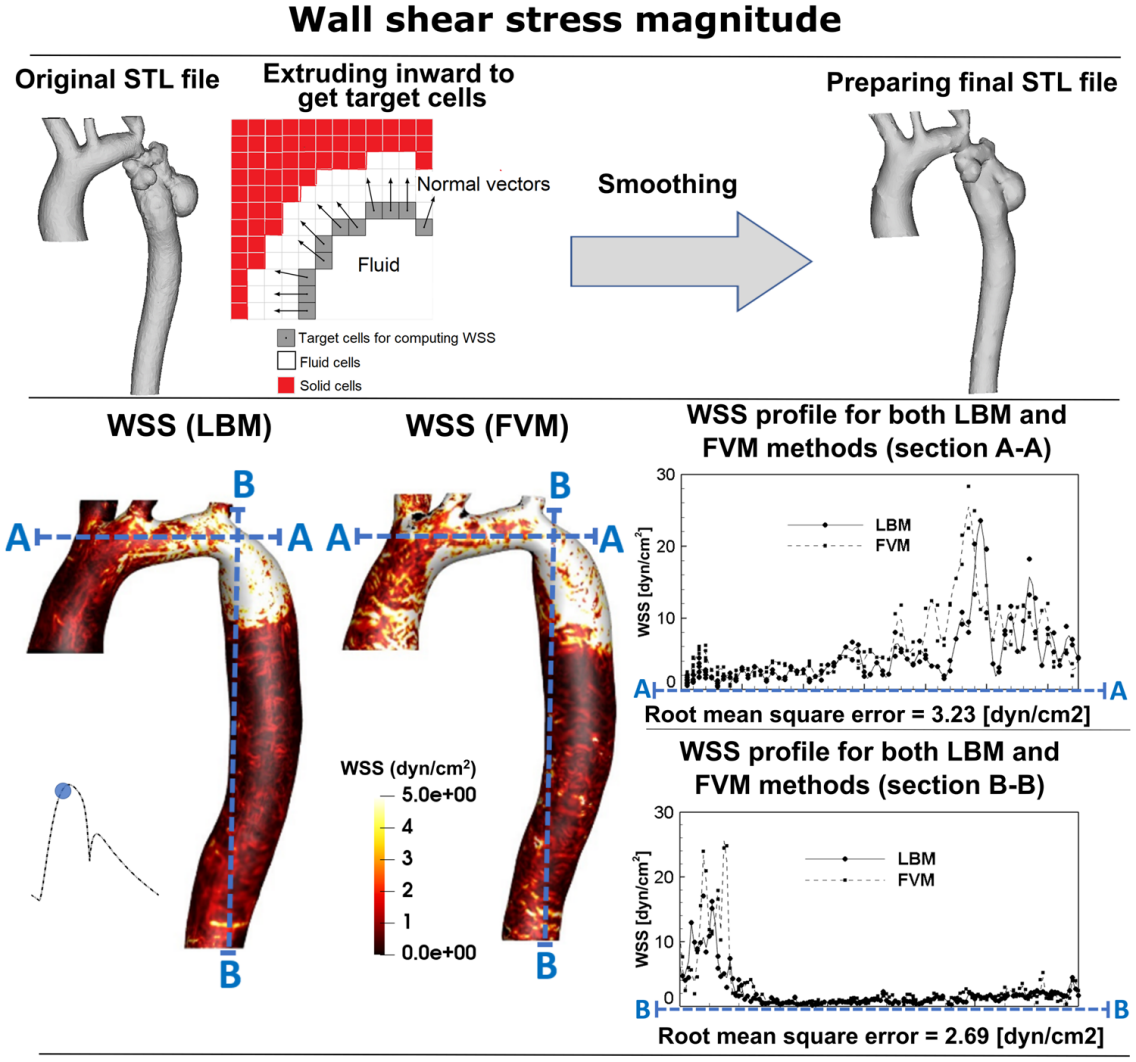
*Wall shear stress comparison*. Wall shear stress (WSS) through the aorta, simulated using LBM and FVM.

**Figure 5:**
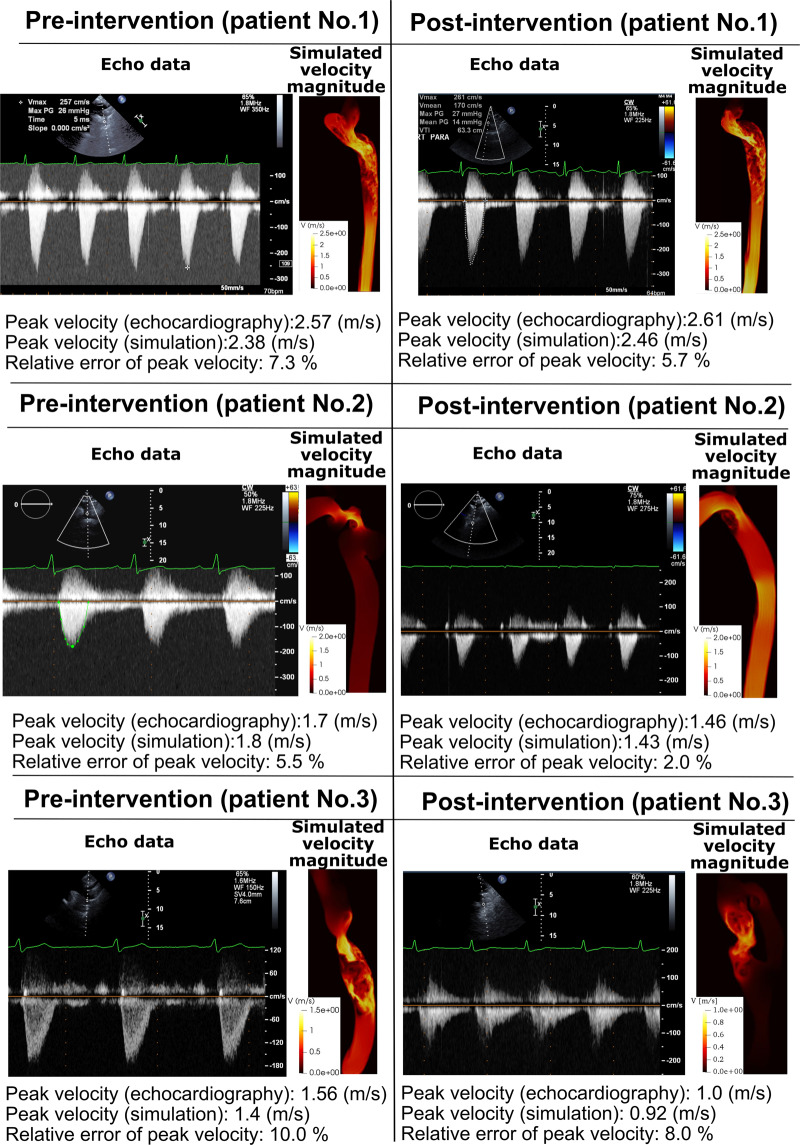
*Validation against Doppler echocardiography*. Doppler echocardiography data and results of the computational framework (based on LPM and LBM) in Patients No. 1 to 3 in pre and post intervention status.

We developed a fast computational fluid dynamics framework to simulate local and global hemodynamics in patients with COA in both pre- and post-intervention states (Fig. 1, schematic diagram). This framework is based on lumped parameter modeling^11,31,32^ and 3-D LBM (LES, Smagorinsky subgrid scale model) as implemented in the open-source OpenLB library^33^ with some supplements as explained below. Table 1 compares the computation time for LBM and finite volume method (FVM) in similar patients and shows that days of calculations were shortened to few hours of calculation using our framework. Calculations of this computational fluid dynamics framework were validated against clinical cardiac catheterization data (Fig. 2), LES calculations using conventional finite-volume method (Figs. 3 and 4) and Doppler echocardiographic measurements (Fig. 5).

**Table 1:** Computation time.

Cases		Wall time	
		LBM	FVM
Benchmark		1^*H*^6^*M*^	2^*D*^20^*H*^
Patient #1	Pre-intervention	1^*H*^41^*M*^	3^*D*^2^*H*^
	Post-intervention	2^*H*^36^*M*^	3^*D*^10^*H*^
Patient #2	Pre-intervention	1^*H*^49^*M*^	4^*D*^14^*H*^
	Post-intervention	1^*H*^22^*M*^	3^*D*^3^*H*^
Patient #3	Pre-intervention	1^*H*^58^*M*^	4^*D*^3^*H*^
	Post-intervention	2^*H*^13^*M*^	5^*D*^5^*H*^

Note: “D”: day, “H”: hour, “M”: Minute.

Computation time on 24 Intel X5650@2.67 GHz cores for both LBM and FVM simulations for all patients investigated in this study in both pre and post intervention states. FVM (OpenFOAM) solver was based on the PISOFOAM method and dynamicEqn LES model, with the minimum resolution of 6.0 × 10 −5 (m) and the temporal resolution of 5.0 × 10 −4 (s).

### Lattice Boltzmann Method (LBM)

The blood flow is mostly laminar in healthy vascular system, while under pathophysiological conditions, the blood flow becomes turbulent distally. Approaches based on the Reynolds-averaged Navier Stokes (RANS) equations are the most prevalent to model but with noticeable limitations to model pulsatile flows^34^. Direct numerical simulations (DNS) tax computing resources and are restricted to low Reynolds numbers. Large eddy simulation (LES) approach, which sits between DNS and RANS, is a technique well suited for the computational modeling of turbulent vascular flows with a high potential in modeling the physiological low-Reynolds transitional flows^11^. Although the conventional LES has allowed turbulent modeling, it is still computationally expensive. To compensate this, here we used a rather fast 3-D LBM-based computational fluid dynamics approach using LES (Smagorinsky subgrid scale model) to simulate blood flow through the vascular system.

#### Governing equations

The simplest form of LBM equations is based on *Bhatnagar-Gross-Krook* (BGK) approximation with single relaxation time^35^. The discretized form of Boltzmann equation based on BGK approximation is as follows^36^:

1 $${{\rm{f}}}_{{\rm{\alpha }}}({\rm{x}}+{{\rm{e}}}_{{\rm{\alpha }}}{\rm{\delta }}{\rm{t}},{\rm{t}}+{\rm{\delta }}{\rm{t}})-{{\rm{f}}}_{{\rm{\alpha }}}({\rm{x}},{\rm{t}})=-\,1/{\rm{\tau }}({{\rm{f}}}_{{\rm{\alpha }}}({\rm{x}},{\rm{t}})-{{\rm{f}}}_{{\rm{\alpha }}}^{{\rm{e}}{\rm{q}}}({\rm{x}},{\rm{t}})$$

For BGK-LBM model with *Q* velocities, a set of distribution functions {f_α_|α = 0,1, …, Q − 1} is defined on each lattice node (x). τ, t and f^eq^ are relaxation time, discrete time and Maxwell-Boltzmann equilibrium distribution function, respectively. Note that subscript α depends on the number of lattice vectors.

**Figure 6:**
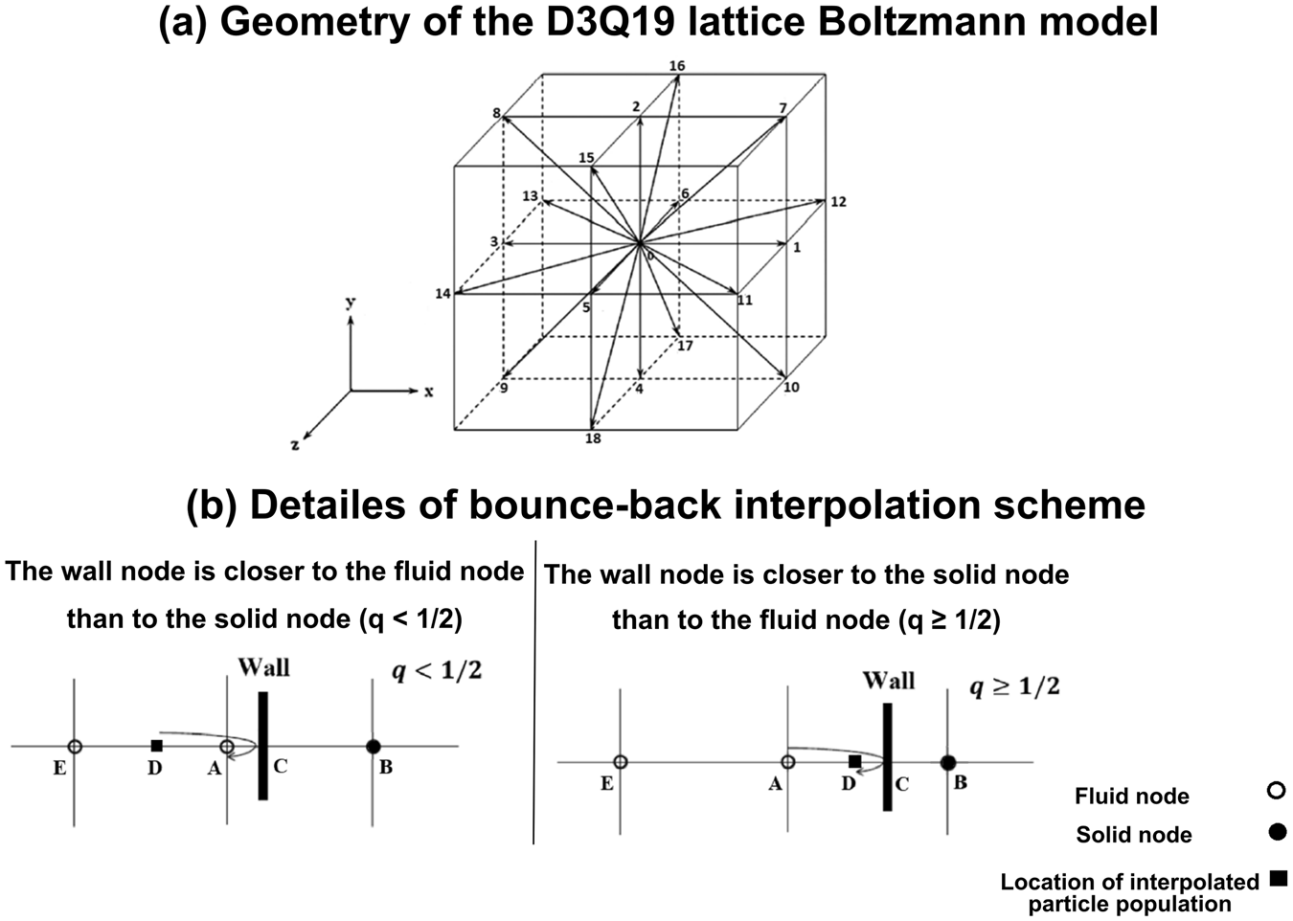
*Modeling complex geometries in LBM*. (**a**) Geometry of D3Q19 discrete velocity model with lattice vectors of e_i_ (Eq. 2); (**b**) Details of bounce-back interpolation scheme (Here A and E are fluid nodes, B is solid nodes and D represents the location of an interpolated population): (I) The wall-node C is closer to the fluid-node A than to the solid-node B (q < 1/2). In this case, interpolations are required to construct post collision state at node D. We constructed the unknown quantities at node A from particles population at node D that will travel to node A after bouncing back off the wall. (II) The wall-node *C* is closer to the solid-node *B* than to the fluid-node *A* (*q*≥1/2). In this case, endpoint of propagation state (node D) lies between the boundary node (A) and the wall node (C) and the information of the particle leaving node A and arriving node D will be used to compute the unknown quantities at node A^42,74,75^.

The LBM follows D_x_Q_y_ reference in which x and y are number of dimensions and number of particle velocities, respectively. In this study, we considered D_3_Q_19_, referred to the three-dimensional nineteen-velocity model, to simulate blood flow across the aorta (Fig. 6, Panel A). The discrete velocity vectors in D_3_Q_19_ is as follows^37^:

$${{\rm{e}}}_{0},{{\rm{e}}}_{1},{{\rm{e}}}_{2},{{\rm{e}}}_{3},{{\rm{e}}}_{4},{{\rm{e}}}_{5},{{\rm{e}}}_{6},{{\rm{e}}}_{7},{{\rm{e}}}_{8},{{\rm{e}}}_{9},{{\rm{e}}}_{10},{{\rm{e}}}_{11},{{\rm{e}}}_{12},{{\rm{e}}}_{13},{{\rm{e}}}_{14},{{\rm{e}}}_{15},{{\rm{e}}}_{16},{{\rm{e}}}_{17},{{\rm{e}}}_{18}\,=$$

2 $$[\begin{array}{ccc}\begin{array}{ccc}0 & 1 & 0\\ 0 & 0 & 1\\ 0 & 0 & 0\end{array} & \begin{array}{ccc}-1 & 0 & 0\\ 0 & -1 & 0\\ 0 & 0 & 1\end{array} & \begin{array}{ccc}\begin{array}{ccc}0 & 1 & -1\\ 0 & 1 & 1\\ -1 & 0 & 0\end{array} & \begin{array}{c}-1\\ -1\\ 0\end{array} & \begin{array}{ccc}\begin{array}{c}1\\ -1\\ 0\end{array} & \begin{array}{c}1\\ 0\\ 1\end{array} & \begin{array}{ccc}\begin{array}{c}1\\ 0\\ -1\end{array} & \begin{array}{ccc}-1 & -1 & 0\\ 0 & 0 & 1\\ -1 & 1 & 1\end{array} & \begin{array}{cc}\begin{array}{c}0\\ 1\\ -1\end{array} & \begin{array}{cc}\begin{array}{c}0\\ -1\\ -1\end{array} & \begin{array}{c}0\\ -1\\ 1\end{array}\end{array}\end{array}\end{array}\end{array}\end{array}\end{array}]$$

For the lattice speed of sound ${{\rm{c}}}_{{\rm{s}}}=1/\sqrt{3}$, Maxwell–Boltzmann distribution function (${{\rm{f}}}_{{\rm{\alpha }}}^{{\rm{e}}{\rm{q}}}$) is defined as follows^38^:

3 $${{\rm{f}}}_{{\rm{\alpha }}}^{{\rm{e}}{\rm{q}}}={{\rm{w}}}_{{\rm{\alpha }}}{\rm{\rho }}\left[1+\frac{{{\rm{e}}}_{{\rm{\alpha }}}{\rm{.u}}}{{{\rm{c}}}_{{\rm{s}}}^{2}}+\frac{{({{\rm{e}}}_{{\rm{\alpha }}}{\rm{.u}})}^{2}}{2{{\rm{c}}}_{{\rm{s}}}^{4}}-\frac{({\rm{u}}{\rm{.u}})}{2{{\rm{c}}}_{{\rm{s}}}^{2}}\right]$$

In Eq. (3), u is velocity, w_α_ is the weighting coefficients which is given by w_0_ = 1/3, w_1~6_ = 2/36 and w_7~18_ = 1/36 for D_3_Q_19_ model, e_α_ is the discrete velocity vector in α direction (α = 0, …, 18) and ρ is the lattice density.

In this study, a multi-relaxation time (MRT) LBM-based model was implemented to overcome some defects of BGK model such as fixed ratio of kinematic and bulk viscosities as well as fixed Prandtl number which cause instabilities at high Reynolds numbers^39^. In this regard, Eq. (1) was modified to Eq. (4) considering MRT scheme as follows:

4 $${{\rm{f}}}_{{\rm{\alpha }}}({{\rm{x}}+{\rm{e}}}_{{\rm{\alpha }}}{\rm{\delta }}{\rm{t}},{\rm{t}}+{\rm{\delta }}{\rm{t}}){-{\rm{f}}}_{{\rm{\alpha }}}({\rm{x}},{\rm{t}}){\,=\,-{\rm{M}}}_{{\rm{\alpha }}{\rm{\gamma }}}^{-1}{\hat{{\rm{S}}}}_{{\rm{\gamma }}{\rm{k}}}{({\rm{m}}}_{{\rm{k}}}({\rm{x}},{\rm{t}}){\,-\,{\rm{m}}}_{{\rm{k}}}^{{\rm{eq}}}({\rm{x}},{\rm{t}})$$

where, m_k_(x,t) and m_k_^eq^(x,t) indicate vectors of moments and their equilibrium functions. M and Ŝ are the transform matrix and collision matrix, respectively.

Mappings between moment and distribution functions were performed by linear transformation as follows:

5 $${\rm{m}}={\rm{M}}.{[{f}_{0},{f}_{2},\ldots ,{f}_{18}]}^{T}\,{\rm{and}}\,[{f}_{0},{f}_{2},\,\ldots ,\,{f}_{18}]={{\rm{M}}}^{-1}{\rm{m}}$$

The Equilibrium distribution function must satisfy conservation of mass and momentum^40^. Therefore, mass and momentum were conserved by Eqs. (6) and (7), respectively:

6 $${\rm{\rho }}=\mathop{\sum }\limits_{{\rm{\alpha }}=0}^{{\rm{\alpha }}=18}{{\rm{f}}}_{{\rm{\alpha }}}^{{\rm{e}}{\rm{q}}}=\mathop{\sum }\limits_{{\rm{\alpha }}=0}^{{\rm{\alpha }}=18}{{\rm{f}}}_{{\rm{\alpha }}}$$

7 $${\rm{{\rm P}}}{\rm{u}}=\mathop{\sum }\limits_{{\rm{\alpha }}=0}^{{\rm{\alpha }}=18}{{\rm{f}}}_{{\rm{\alpha }}}^{{\rm{e}}{\rm{q}}}{{\rm{e}}}_{{\rm{\alpha }}}=\mathop{\sum }\limits_{{\rm{\alpha }}=0}^{{\rm{\alpha }}=18}{{\rm{f}}}_{{\rm{\alpha }}}{{\rm{e}}}_{{\rm{\alpha }}}$$

The transformation matrix M for D3Q19 is defined as the following:

8 $${\rm{M}}=[\begin{array}{ccccccccccccccccccc}1 & 1 & 1 & 1 & 1 & 1 & 1 & 1 & 1 & 1 & 1 & 1 & 1 & 1 & 1 & 1 & 1 & 1 & 1\\ -3 & -11 & -11 & -11 & -11 & -11 & -11 & 8 & 8 & 8 & 8 & 8 & 8 & 8 & 8 & 8 & 8 & 8 & 8\\ 12 & -4 & -4 & -4 & -4 & -4 & -4 & 1 & 1 & 1 & 1 & 1 & 1 & 1 & 1 & 1 & 1 & 1 & 1\\ 0 & 1 & -1 & 0 & 0 & 0 & 0 & 1 & -1 & 1 & -1 & 1 & -1 & 1 & -1 & 0 & 0 & 0 & 0\\ 0 & -4 & 4 & 0 & 0 & 0 & 0 & 1 & -1 & 1 & -1 & 1 & -1 & 1 & -1 & 0 & 0 & 0 & 0\\ 0 & 0 & 0 & 1 & -1 & 0 & 0 & 1 & 1 & -1 & -1 & 0 & 0 & 0 & 0 & 1 & -1 & -1 & -1\\ 0 & 0 & 0 & -4 & 4 & 0 & 0 & 1 & 1 & -1 & -1 & 0 & 0 & 0 & 0 & 1 & -1 & -1 & -1\\ 0 & 0 & 0 & 0 & 0 & 1 & -1 & 0 & 0 & 0 & 0 & 1 & 1 & -1 & -1 & 1 & 1 & -1 & -1\\ 0 & 0 & 0 & 0 & 0 & -4 & 4 & 0 & 0 & 0 & 0 & 1 & 1 & -1 & -1 & 1 & 1 & -1 & -1\\ 0 & 2 & 2 & -1 & -1 & -1 & -1 & 1 & 1 & 1 & 1 & 1 & 1 & 1 & 1 & -2 & -2 & -2 & -2\\ 0 & -4 & -4 & 2 & 2 & 2 & 2 & 1 & 1 & 1 & 1 & 1 & 1 & 1 & 1 & -2 & -2 & -2 & -2\\ 0 & 0 & 0 & 1 & 1 & -1 & -1 & 1 & 1 & 1 & 1 & -1 & -1 & -1 & -1 & 0 & 0 & 0 & 0\\ 0 & 0 & 0 & -2 & -2 & 2 & 2 & 1 & 1 & 1 & 1 & -1 & -1 & -1 & -1 & 0 & 0 & 0 & 0\\ 0 & 0 & 0 & 0 & 0 & 0 & 0 & 1 & -1 & -1 & 1 & 0 & 0 & 0 & 0 & 0 & 0 & 0 & 0\\ 0 & 0 & 0 & 0 & 0 & 0 & 0 & 0 & 0 & 0 & 0 & 0 & 0 & 0 & 0 & 1 & -1 & 1 & 1\\ 0 & 0 & 0 & 0 & 0 & 0 & 0 & 0 & 0 & 0 & 0 & 1 & -1 & -1 & 1 & 0 & 0 & 0 & 0\\ 0 & 0 & 0 & 0 & 0 & 0 & 0 & -1 & -1 & 1 & -1 & -1 & 1 & -1 & 1 & 0 & 0 & 0 & 0\\ 0 & 0 & 0 & 0 & 0 & 0 & 0 & -1 & -1 & 0 & 1 & 0 & 0 & 0 & 0 & 1 & -1 & -1 & -1\\ 0 & 0 & 0 & 0 & 0 & 0 & 0 & 0 & 0 & 0 & 0 & 1 & 1 & -1 & -1 & -1 & -1 & -1 & 1\end{array}]$$

The corresponding macroscopic moments vector are:

9 $${{\rm{m}}}_{\alpha }={({{\rm{m}}}_{0},{{\rm{m}}}_{1},\ldots ,{{\rm{m}}}_{18})}^{{\rm{T}}}$$

Diagonal matrix Ŝ in Eq. (4) is defined as follows:

10 $$\hat{{\rm{S}}}={\rm{diag}}\,(0,1.19,\,1.4,0,1.2,0,\,1.2,{\rm{v}},1.4,{\rm{v}},{\rm{v}},{\rm{v}},1.98,1.98,1.98)$$

where v is physical viscosity and (m^eq^) is equilibrium moments matrix, as shown below:

11 $${{\rm{m}}}_{{\rm{k}}}^{{\rm{eq}}}={({{{\rm{m}}}_{0}}^{{\rm{eq}}}{{,{\rm{m}}}_{1}}^{{\rm{eq}}}{,{\rm{\ldots }},{\rm{m}}}_{18}^{{\rm{eq}}})}^{{\rm{T}}}$$

The equilibrium moments in Eq. (11) were obtained as follows:

12 $${{\rm{m}}}_{0}^{{\rm{eq}}}={{\rm{\rho }},\,{\rm{m}}}_{1}^{{\rm{eq}}}=-11{\rm{\rho }}+\frac{19}{{\rm{\rho }}}({{{\rm{j}}}_{{\rm{x}}}}^{2}{{+{\rm{j}}}_{{\rm{y}}}}^{2}{{+{\rm{j}}}_{{\rm{z}}}}^{2}),\,{{\rm{m}}}_{2}^{{\rm{eq}}}=\frac{-475}{63}\frac{1}{{\rm{\rho }}}({{{\rm{j}}}_{{\rm{x}}}}^{2}{{+{\rm{j}}}_{{\rm{y}}}}^{2}{{+{\rm{j}}}_{{\rm{z}}}}^{2})$$

13 $${{\rm{m}}}_{3}^{{\rm{eq}}}={{\rm{j}}}_{{\rm{x}}},\,{{\rm{m}}}_{4}^{{\rm{eq}}}=-\frac{2}{3}{{\rm{j}}}_{{\rm{x}}},\,{{\rm{m}}}_{5}^{{\rm{eq}}}={{\rm{j}}}_{{\rm{y}}}{,\,{\rm{m}}}_{6}^{{\rm{eq}}}=-\frac{2}{3}{{\rm{j}}}_{{\rm{y}}}{,\,{\rm{m}}}_{7}^{{\rm{eq}}}={{\rm{j}}}_{{\rm{z}}},$$

14 $${{\rm{m}}}_{8}^{{\rm{e}}{\rm{q}}}=-\frac{2}{3}{{\rm{j}}}_{{\rm{z}}},{{\rm{m}}}_{9}^{{\rm{e}}{\rm{q}}}=\frac{1}{{\rm{\rho }}}[2{{\rm{j}}}_{{\rm{x}}}^{2}-({{\rm{j}}}_{{\rm{y}}}^{2}+{{\rm{j}}}_{{\rm{z}}}^{2})],\,$$

15 $${{\rm{m}}}_{10}^{{\rm{eq}}}=0,\,{{\rm{m}}}_{11}^{{\rm{eq}}}=\frac{1}{{\rm{\rho }}}[{{\rm{j}}}_{{\rm{y}}}^{2}{-{\rm{j}}}_{{\rm{z}}}^{2}],\,{{\rm{m}}}_{12}^{{\rm{eq}}}=0,$$

16 $${{\rm{m}}}_{13}^{{\rm{e}}{\rm{q}}}=\frac{1}{{\rm{\rho }}}{{\rm{j}}}_{{\rm{x}}}{{\rm{j}}}_{{\rm{y}}},\,{{\rm{m}}}_{14}^{{\rm{e}}{\rm{q}}}=\frac{1}{{\rm{\rho }}}{{\rm{j}}}_{{\rm{y}}}{{\rm{j}}}_{{\rm{z}}},\,{{\rm{m}}}_{15}^{{\rm{e}}{\rm{q}}}=\frac{1}{{\rm{\rho }}}{{\rm{j}}}_{{\rm{x}}}{{\rm{j}}}_{{\rm{z}}},$$

17 $${m}_{16}^{eq}={m}_{17}^{eq}={m}_{18}^{eq}=0,$$

The momentum j = (j_x_, j_y_, j_z_) was defined as follows:

18 $${{\rm{j}}}_{{\rm{x}}}={{\rm{\rho }}{\rm{u}}}_{{\rm{x}}},\,{{\rm{j}}}_{{\rm{y}}}={{\rm{\rho }}{\rm{u}}}_{{\rm{y}}},\,{{\rm{j}}}_{{\rm{z}}}={{\rm{\rho }}{\rm{u}}}_{{\rm{z}}}$$

#### Lattice boltzmann method & large eddy simulation

In this study, turbulent modeling was performed via Large Eddy Simulation employing Smagorinsky subgrid scale model. The physical viscosity is a superposition of the molecular kinematic viscosity (v_mol_) and turbulent viscosity (v_turb_), related to the length scale or lattice size (Δ_x_). Collision time (τ) was therefore changed as the following^41^:

19 $${\rm{\tau }}={{\rm{\tau }}}_{{\rm{mol}}}\,+\,{{\rm{\tau }}}_{{\rm{turb}}}$$

The molecular and turbulent collision time in Eq. (19) were obtained as the following:

20 $${{\rm{\tau }}}_{{\rm{mol}}}={{\rm{3v}}}_{{\rm{mol}}}+0.5$$

21 $${{\rm{\tau }}}_{{\rm{turb}}}=0.5(\sqrt{{{{\rm{\tau }}}_{{\rm{mol}}}}^{2}+\left({({{\rm{C}}}_{s}{\Delta }_{x})}^{2}\frac{{\Delta }_{t}}{{{\rm{C}}}_{s}}4\sqrt{2}\tau \bar{\Pi }\right)}-{{\rm{\tau }}}_{{\rm{mol}}}$$

C_s_ and $\bar{\Pi }$ are Smagorinsky constant and second-order moment of the non-equilibrium term of the distribution functions, respectively. Total viscosity, v, is given as^41^:

22 $${\rm{v}}={{\rm{v}}}_{{\rm{mol}}}{+{\rm{v}}}_{{\rm{turb}}}=\frac{1}{3}\left({\rm{\tau }}-\frac{1}{2}\right){{\rm{c}}}^{2}{{\rm{\delta }}}_{{\rm{t}}}=\frac{1}{3}\left({{\rm{\tau }}}_{{\rm{mol}}}+{{\rm{\tau }}}_{{\rm{turb}}}-\frac{1}{2}\right){{\rm{c}}}^{2}{{\rm{\delta }}}_{{\rm{t}}}$$

#### Modeling surface curvature near the wall of complex geometries

An interpolated bounce-back scheme proposed by Bouzidi *et al*.^42^ was used to treat boundaries of inclined and complicated geometries. In this technique, for evaluating the post-propagation state of a fluid node A, next to a curved solid wall, the distribution function (Fig. 6, Panel B) was defined as:

23 $${{f}}_{\bar{{\rm{\alpha }}}}({{x}}_{{A}},{\rm{t}}+\Delta {\rm{t}})=\{\begin{array}{c}\begin{array}{cc}2{\rm{q}}{{\rm{f}}}_{{\rm{\alpha }}}^{{c}}({{x}}_{{A}},{\rm{t}})-(1-2{\rm{q}}){{\rm{f}}}_{{\rm{\alpha }}}^{{c}}({{x}}_{{E}},{\rm{t}}) & {\rm{q}} < \frac{1}{2}\end{array}\\ \begin{array}{cc}\frac{1}{2{\rm{q}}}{{\rm{f}}}_{{\rm{\alpha }}}^{{c}}({{x}}_{{A}},{\rm{t}})+\frac{(2{\rm{q}}-1)}{2{\rm{q}}}{{\rm{f}}}_{{\rm{\alpha }}}^{{c}}({{x}}_{{A}},{\rm{t}}) & {\rm{q}}\ge \frac{1}{2}\end{array}\end{array}$$

where ${{f}}_{\bar{{\rm{\alpha }}}}({{x}}_{{A}},{\rm{t}}+\Delta {\rm{t}})$ is the post-collision and post-propagation state of the distribution function at point *x*_*A*_ and time (t + Δt) and f_α_^c^ is the value of distribution function after a collision and before propagation state of the fluid node. The factor q is the normalized distance from the wall which equals to $\frac{|{\rm{AC}}|}{|{\rm{AB}}|}$ (Fig. 6, Panel B, schematic diagram for one dimensional problem).

#### Wall shear stress

Wall shear stress (WSS) is a frictional force induced by fluid moving along a solid wall. The total stress tensor for the fluid is as the following:

24 $${T}_{ij}=-p.{\delta }_{ij}+{\sigma }_{ij}$$

where *p*, *δ*_*ij*_ and *σ*_*ij*_ are pressure, Kronecker symbol and contribution from the viscous force. The stress on boundary surface element with normal vector $\overrightarrow{n}$ is *T*_*ij*_*n*_*j*_. The wall stress vector $\overrightarrow{\tau }$ is computed as:

25 $${\tau }_{i}={T}_{ij}{n}_{j}-({n}_{j}{T}_{kj}{n}_{k}){n}_{i}$$

The total stress *T*_*ij*_ can be replaced by *σ*_*ij*_, since the projection of normal stress (*p*.*δ*_*ij*_) on the tangential plane is zero. For a Newtonian fluid, the viscouse stress is proportional to the strain rate tensor (*ε*_*ij*_)^43,44^:

26 $${\sigma }_{ij}=2\mu {\varepsilon }_{ij}=[\begin{array}{ccc}{\tau }_{xx} & {\tau }_{xy} & {\tau }_{xz}\\ {\tau }_{yx} & {\tau }_{yy} & {\tau }_{yz}\\ {\tau }_{zx} & {\tau }_{zy} & {\tau }_{zz}\end{array}]=\left[\begin{array}{ccc}2\mu \frac{\partial u}{\partial x} & \mu \left(\frac{\partial u}{\partial y}+\frac{\partial v}{\partial x}\right) & \mu \left(\frac{\partial u}{\partial z}+\frac{\partial w}{\partial x}\right)\\ \mu \left(\frac{\partial u}{\partial y}+\frac{\partial v}{\partial x}\right) & 2\mu \frac{\partial v}{\partial y} & \mu \left(\frac{\partial v}{\partial z}+\frac{\partial w}{\partial y}\right)\\ \mu \left(\frac{\partial u}{\partial z}+\frac{\partial w}{\partial x}\right) & \mu \left(\frac{\partial v}{\partial z}+\frac{\partial w}{\partial y}\right) & 2\mu \frac{\partial w}{\partial z}\end{array}\right]$$

where (u, v, w) and *μ* are velocity components in three-dimensional coordinates and constant dynamic viscosity, respectively^45^.

To supplement OpenLB calculations, we used finite difference method to compute WSS as follows. The derivatives of the velocity field and consequently the nine WSS tensor components (Eq. 26) were computed using a first-order accuracy finite difference scheme. When estimating a smooth curved boundary by a series of staircases, the LBM captures the coarseness of this approximation and indeed generates a flow field different from the one produced by a smooth boundary. However, such a difference mainly impacts the thin layer close to the boundary because the roughness of the staircase wall can be considered smooth at a distance far enough from the boundary. Measurements of the WSS should be performed at the borders of this boundary layer, and not on cells which directly represent the aorta wall. To improve the accuracy of the WSS, we computed the velocity gradient and normal vectors (Eq. 26) at a few lattice nodes away from the aorta wall, as proposed by the staircase approximation of boundaries method^45^ (Fig. 4). Additionally, we calculated WSS using the distribution function, as customarily done in LBM studies, and observed negligible differences with the WSS calculated with the above described method.

#### Model properties & Boundary conditions

Blood was assumed to be a Newtonian and incompressible fluid with dynamic viscosity of 0.0035 Pa·s and density of 1050 kg/m^3^. Aortic local flow dynamics is greatly influenced by upstream and downstream flow conditions and the correct choice of boundary conditions is crucial as it chiefly affects the accuracy of the flow simulations. A lumped-parameter model (Fig. 1; see below for details of the lumped parameter model), simulated the function of the left side of the heart was used to impose the time-dependent inlet flow at the ascending aorta position and the outlet pressure at the descending aorta position. We assumed that the flow at the inlet has a Poiseuille flow profile and the time-dependent flow rate obtained from the lumped-parameter model was used to scale this profile to realize this time-varying inlet boundary condition^25,26,46^. The inlet velocity boundary condition in lattice Boltzmann was implemented using the method suggested by Skordos^46^, which uses a second-order finite difference scheme to compute the velocity gradient at the boundary nodes and extrapolates the pressure distribution at the inlet from bulk nodes^47^. Furthermore, in order to avoid pressure fluctuation artifacts at the inlet, a sinusoidal smooth start-up phase was used to the initiate the simulation and smoothly increase velocity from zero initial conition^25,26^. The total flow rate going to the branches was calculated using the lumped-parameter model and was distributed to the branches based on their relative cross-sectional areas at the inlet of each branch. Note our lumped parameter model used a limited number of input parameters that all can be reliably measured using Doppler echocardiography and a sphygmomanometer. No-slip boundary condition was applied at the solid walls as described above (Section: *Modeling surface curvature near the wall of complex geometries*). The aortic wall was treated as a rigid wall as Jin *et al*.^48^ and Keshavarz-Motamed *et al*.^11,16,32^ showed that rigid-wall assumption for the aorta is reasonable and as patients with COA are usually hypertensive and characterized by reduced compliance and elevated stiffness in both proximal and distal aorta, e.g., ^49–51^.

#### Reconstructed geometries in patients with coarctation

We used CT images for patients with coarctation of the aorta to segment and reconstruct the 3D geometries of the complete aorta including ascending aorta, aortic branches and descending aorta using ITK-SNAP (version 3.8.0-BETA), a 3-D image processing and model generation software package (Fig. 1). These 3-D reconstructions were voxelized into multiblocks. Blocks were distributed between computer processor units in order to parallelize the simulation.

#### Numerical strategy

Multiple relaxation time (MRT) LBM-based model was coupled with Smagorinsky turbulent model in order to stabilize complex turbulent fluid flow across the domain. For treating complex geometry, we utilized second order accuracy method proposed by Bouzidi *et al*.^42^. In order to suppress the undesired pressure fluctuation, a smooth startup phase was added to the inlet velocity condition. For turbulent modelling, Large Eddy Smagorinsky subgrid-scale model with constant *C*_*s*_ = 0.1 was applied^41^. Mesh independency was judged by two criteria: velocity and wall shear stress. Mesh definition was considered acceptable if no significant differences (lower than 5%) between successive mesh refinements were noticed in both wall shear stress and velocity fields. The non-dimensional wall distance *y* ^+^ was less than 1, which ensured that the near-wall resolution was fine enough, and turbulence effects were resolved accurately.

### Lumped parameter model

We developed a patient-specific lumped-parameter model, described in details elsewhere^11,16,31,32^, that considers interactions of the aortic valve, LV, COA and arterial system to estimate the flow and pressure through circulatory system as well as the LV function non-invasively (Fig. 1, schematic diagram; Table 2, parameters used in the model) in both pre and post intervention conditions. The model used a limited number of input parameters that can be reliably measured using Doppler echocardiography and a sphygmomanometer. Doppler echocardiography-based parameters (e.g., stroke volume, heart rate, ejection time, ascending aorta area, aortic valve effective orifice area and aortic regurgitation effective orifice area) were measured in the parasternal long axis, parasternal short axis, apical two-chamber, apical four-chamber, and apical five-chamber views of the heart. Other input parameters of the model were systolic and diastolic blood pressures measured using a sphygmomanometer. Note that the proposed method does not need any catheter data as input parameters to the model. The model and sub-models have already been used and validated against *in vivo* cardiac catheterization and *in vivo* MRI data^11,16,31,32^.

**Table 2:** Summarized cardiovascular parameters used in the lumped parameter modeling to simulate all cases.

Description	Abbreviation	Value
**COA and valve parameters**		
Effective orifice area	EOA	From echocardiography data
Energy loss coefficient	E_L_Co	$\frac{(EOA)A}{A-EOA}$ From echocardiography data
Variable resistance	R_coa_, R_av_ and R_ar_	$\frac{\rho }{2{E}_{L}C{o}^{2}}Q$
Inductance	L_coa_, L_av_ and L_ar_	$\frac{2\pi \rho }{\sqrt{{E}_{L}Co}}$
**Systematic circulation parameters**		
Aortic resistance	R_ao_	0.05 mmHg.s.mL^−1^
Aortic compliance	C_ao_	Initial value: 0.5 mL/mmHg; Adjusted for each degree of hypertension (Proximal COA compliance)
Systemic vein resistance	R_SV_	0.05 mmHg.s.mL^−1^
Systemic arteries and veins compliance	C_SAC_	Initial value: 2 mL/mmHg; Adjusted for each degree of hypertension (Systemic compliance)
systemic arteries resistance (including arteries, arterioles and capillaries)	R_SA_	0.8 mmHg.s.mL^−1^ ; Adjusted according to the calculated total systemic resistance
Proximal descending aorta resistance	R_pda_	0.05 mmHg·s·mL^−1^
Upper body resistance	R_ub_	Adjusted to have 15% of total flow rate in healthy case
**Output condition**		
Central venous pressure	P_CV0_	4 mmHg
**Input condition**		
Mitral valve mean flow rate	Q_mv_	From echocardiography data
**Other**		
Constant blood density		1050 kg/m^3^
Heart rate	HR	From echocardiography data
Duration of cardiac cycle	T	From echocardiography data

#### Heart-arterial model

The ventricle was filled by a normalized physiological mitral flow waveform adjusted for the required stroke volume. Coupling between LV pressure and volume was performed using a time varying elastance E(t), a measure of cardiac muscle stiffness.

27 $$E(t)=\frac{{P}_{LV}(t)}{V(t)-{V}_{0}}$$

where P_LV_(t), V(t) and V_0_ are left ventricular time-varying pressure, time-varying volume and unloaded volume, respectively. The amplitude of E(t) was normalized with respect to maximal elastance E_max_, *i.e*., the slope of the end-systolic pressure-volume relation, giving E_N_(t_N_) = E(t)/E_max_. Time was normalized with respect to the time to reach peak elastance, T_Emax_ (t_N_ = t/T_Emax_). These normalized time-varying elastance curves E_N_(t_N_) have similar shapes in the normal human heart under numerous inotropic conditions or in affected human hearts irrespective of disease etiology.

28 $${E}_{max}{E}_{N}(t/{T}_{Emax})=\frac{{P}_{LV}(t)}{V(t)-{V}_{0}}$$

This normalized curve can be described mathematically, and therefore, if E_N_(t_N_) is given, the relation between P_LV_(t) and V(t) can be concluded for any LV.

#### Modeling aortic valve

Aortic valve was modeled using the following net pressure gradient formulation across the aortic valve during the LV ejection:

29 $${TP{G}_{net}|}_{av}={P}_{LV}(t)-{P}_{A}(t)=\frac{2\pi \rho }{\sqrt{{{E}_{L}Co|}_{av}}}\frac{\partial Q(t)}{\partial t}+\frac{\rho }{2{{E}_{L}Co|}_{av}^{2}}{Q}^{2}(t)$$

30 $${{E}_{L}Co|}_{av}=\frac{({EOA|}_{av})A}{A-{EOA|}_{av}}$$

where *E*_*L*_*Co*|_*av*_, *EOA*|_*av*_, *A*, *ρ* and *Q* are valvular energy loss coefficient, aortic valve effective orifice area, ascending aorta cross sectional area, fluid density and transvalvular flow rate, respectively.

#### Modeling aortic valve regurgitation

Aortic regurgitation (AR) was modeled using the following formulation. AR pressure gradient is the difference between aortic pressure and LV pressure during diastole.

31 $${TP{G}_{net}|}_{ar}=\frac{2\pi \rho }{\sqrt{{{E}_{L}Co|}_{ar}}}\frac{\partial Q(t)}{\partial t}+\frac{\rho }{2{{E}_{L}Co|}_{ar}^{2}}{Q}^{2}(t)$$

32 $${{E}_{L}Co|}_{ar}=\frac{(REOA){A}_{LVOT}}{{A}_{LVOT}-REOA}$$

where *E*_*L*_*Co*|_*ar*_, *REOA* and *A*_*LVOT*_ are regurgitation energy loss coefficient, regurgitant effective orifice area and LVOT area, respectively.

#### Modeling coarctation of the aorta

The characteristics of the arterial system are important when modeling COA as only a portion of total flow rate will cross the COA. To consider this, two parallel branches were considered: (1) the first branch simulates the flow towards the upper body, or the flow bypassing the COA (including aortic arch arteries and potential collaterals); (2) a second branch simulates the flow crossing COA and directed towards descending aorta. This branch includes a resistance for the proximal descending aorta, and a time-varying resistance and an inductance which together represent the trans-coarctation net pressure gradient induced by the COA:

33 $${TP{G}_{net}|}_{coa}=\frac{2\pi \rho }{\sqrt{{{E}_{L}Co|}_{coa}}}\frac{\partial Q(t)}{\partial t}+\frac{\rho }{2{{E}_{L}Co|}_{coa}^{2}}{Q}^{2}(t)$$

34 $${{E}_{L}Co|}_{COA}=\frac{({EOA|}_{coa})A}{A-{EOA|}_{coa}}$$

where *E*_*L*_*Co*|_*coa*_, *EOA*|_*coa*_, *A*, *ρ* and *Q* are the energy loss coefficient of the COA, the effective orifice area of the COA, aortic cross sectional area downstream of the COA, the fluid density and the trans-coarctation flow rate, respectively. The energy loss coefficient is then described in terms of the aortic cross section just downstream of the COA and the effective orifice area of the COA.

#### Determining arterial compliance and peripheral resistance

The total systemic resistance was computed as the quotient of the average brachial pressure and the cardiac output. This total systemic resistance represents the electrical equivalent resistance for all resistances in the current model. Because what the left ventricle faces is the total systemic resistance and not the individual resistances, we considered the aortic resistance, *R*_*ao*_, and systemic vein resistance, *R*_*SV*_, as constants and adjusted the systemic artery resistance, *R*_*SA*_, according to the obtained total systemic resistance.

For each degree of hypertension, we fit the predicted pulse pressure to the actual pulse pressure (known by arm cuff sphygmomanometer) obtained from clinical study by adjusting compliances (proximal COA (C_ao_) and systemic (C_SAC_)). Therefore, compliance adjustment was done by a simple trial and error for each degree of hypertension.

#### Computational algorithm

A lumped parameter model developed and described in detail elsewhere (7,17,55) was analyzed numerically by creating and solving a system of ordinary differential equations in Matlab Simscape (MathWorks, Inc.), enhanced by adding additional codes to meet demands of cardiac model in circuit. A Fourier series representation of an experimental normalized elastance curve for human adults was used to generate a signal to be fed into the main program. Simulations start at the onset of isovolumic contraction. Left ventricle volume, V(t), is calculated using left ventricle pressure, P_LV_, and time varying elastance values. P_LV_, used in the beginning of calculation, is the initial value assumed across the variable capacitor and is automatically adjusted later by system of equations as solution advances. Left ventricle flow rate subsequently was calculated as time derivative of left ventricle volume. Matlab’s ode23t trapezoidal rule variable-step solver was used to solve system of differential equations with initial time step of 0.1 milliseconds. The convergence residual criterion was set to 10^−5^ and initial voltages and currents of capacitors and inductors set to zero.

### Study population

Three patients with COA who underwent intervention at St. Joseph’s Healthcare and Hamilton Health Sciences (Hamilton, ON, Canada) and Massachusetts General Hospital (Boston, MA, USA)^11^ were retrospectively considered. The protocols were reviewed and approved by the Institutional Review Boards of each institution as follows: the Hamilton Integrated Research Ethics Board (HiREB) of Hamilton Health Sciences and St. Joseph’s Healthcare, both affiliated to McMaster University and the Ethics Committee of Massachusetts General Hospital. Informed consents were obtained from human participants. All methods and measurements were performed in accordance with the relevant guidelines and regulations including guidelines of the American College of Cardiology and American Heart Association.

## Results

### Validation

#### Pressure waveforms

The beat-to-beat pressure calculations of LPM were compared with cardiac catheter pressure measurements in patients investigated in this study. Results of our LPM show good qualitative agreements with cardiac catheter measurements in terms of both shape of the waveform, and specific wave features such as the amplitude and the timing of the systolic peak in the aorta (See Fig. 2 for one example). Note cardiac catheterization is a gold standard in clinics to evaluate hemodynamics, e.g., pressures through the heart and circulatory system. The calculations done by LPM had an average root mean square (RMS) error of 8.6 mmHg in the aorta pressures of the 3 patients in both pre and post intervention states. Moreover, the LPM and its sub models already were validated against *in vivo* cardiac catheterization in patients with COA (N = 34)^11^.

#### Velocity field

Fig. 3 compares examples of the simulated velocity contours calculated using LBM and FVM at different cross sections upstream and downstream of the COA. The results show very good qualitative agreements between LBM and FVM simulation results in all cases. Figure 3 also shows that the velocity profiles calculated using LBM and FVM methods along a diameter upstream and downstream of the COA are in good quantitative agreements with root mean square (RMS) errors between 0.201 and 0.311 m/s. Figure 4 shows good quantitative and qualitative agreements between the instantaneous WSS calculated using the two methods with RMS errors of 3.23 dyn/cm^2^ and 2.69 dyn/cm^2^ for sections A-A and B-B, respectively. Most importantly, the simulated peak velocities downstream of the COA correlated well with Doppler echocardiographic measurements in all 3 patients in both pre and post intervention states with a maximum relative error of 10% (Fig. 5). The good agreements between results calculated using LBM with the ones calculated using FVM and measured using Doppler echocardiography permit us to accept LBM results with confidence to investigate other flow features.

### Aorta fluid dynamics (local hemodynamics)

**Figure 7:**
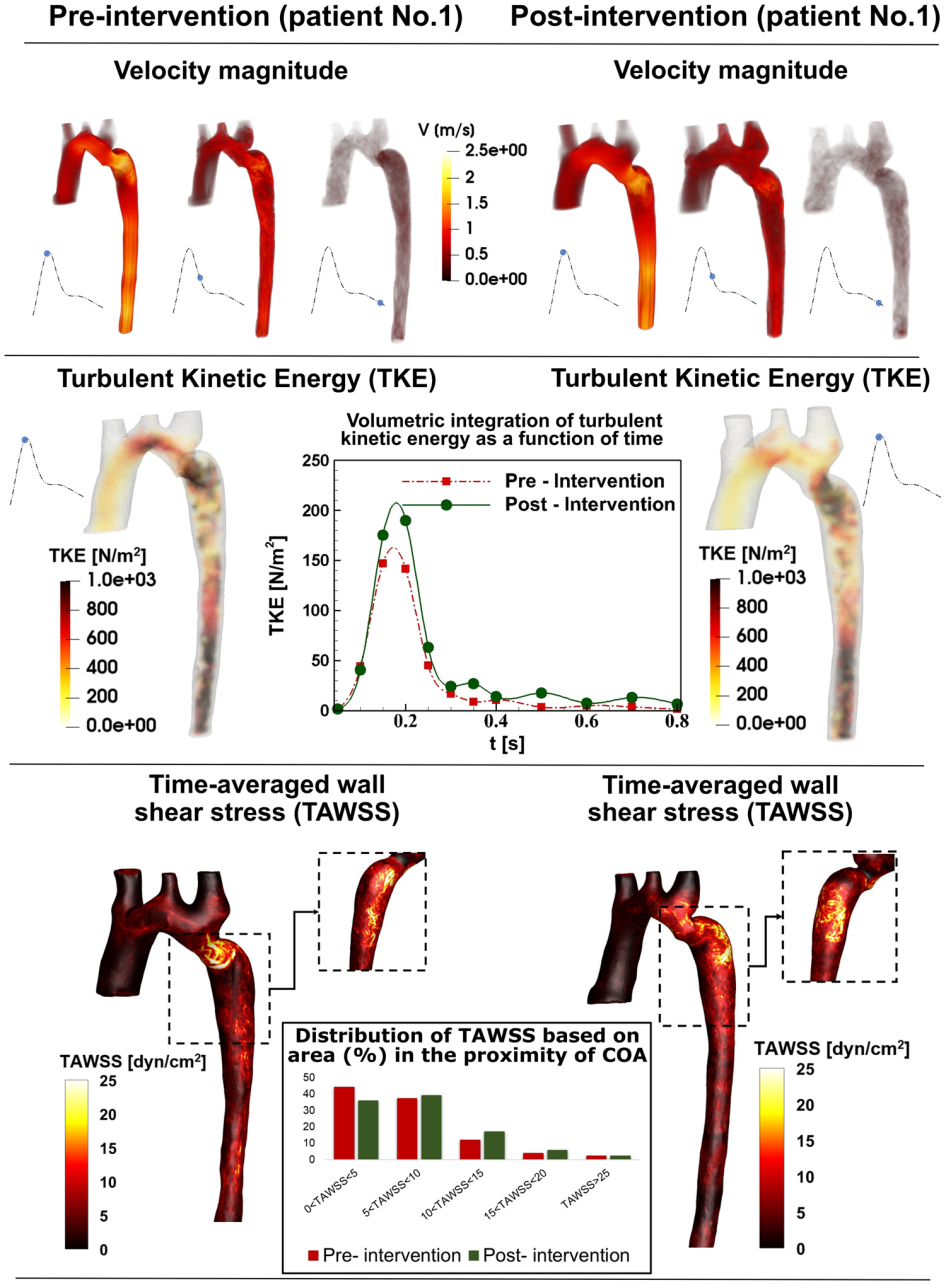
*Flow modeling in Patient No. 1*. Computed velocity magnitude, turbulent kinetic energy (TKE) and time-averaged wall shear stress (TAWSS) using the computational framework (based on LPM and LBM) in pre and post intervention status in Patient No. 1. Patient No. 1 underwent intravascular stent intervention to correct the coarctation. Post intervention, the stent was deployed with residual stenosis due to malapposition of the stent proximal to the coarctation. Angiography post dilatation did not reveal a dissection or extravasation of contrast. The patient tolerated the procedure well without complication. The total shear stress exerted on the wall throughout the cardiac cycle was evaluated using the time-averaged wall shear stress (TAWSS) which is obtained as ${\rm{TAWSS}}=\frac{1}{{\rm{T}}}{\int }_{0}^{{\rm{T}}}|{\rm{\tau }}|{\rm{dt}}$. Here, T and τ are the cardiac cycle period and instantaneous wall shear stress, respectively. Turbulent kinetic energy can be computed as ${\rm{TKE}}=\frac{1}{2}{\rm{\rho }}(\overline{{{\rm{u}}}^{\text{'}2}}+\overline{{{\rm{v}}}^{\text{'}2}}+\overline{{{\rm{w}}}^{\text{'}2}})$ Here u, v, w and ρ correspond to the three components of the instantaneous velocity vector and density, respectively. The bar and prime denote the ensemble averaged and fluctuating components, respectively.

**Figure 8:**
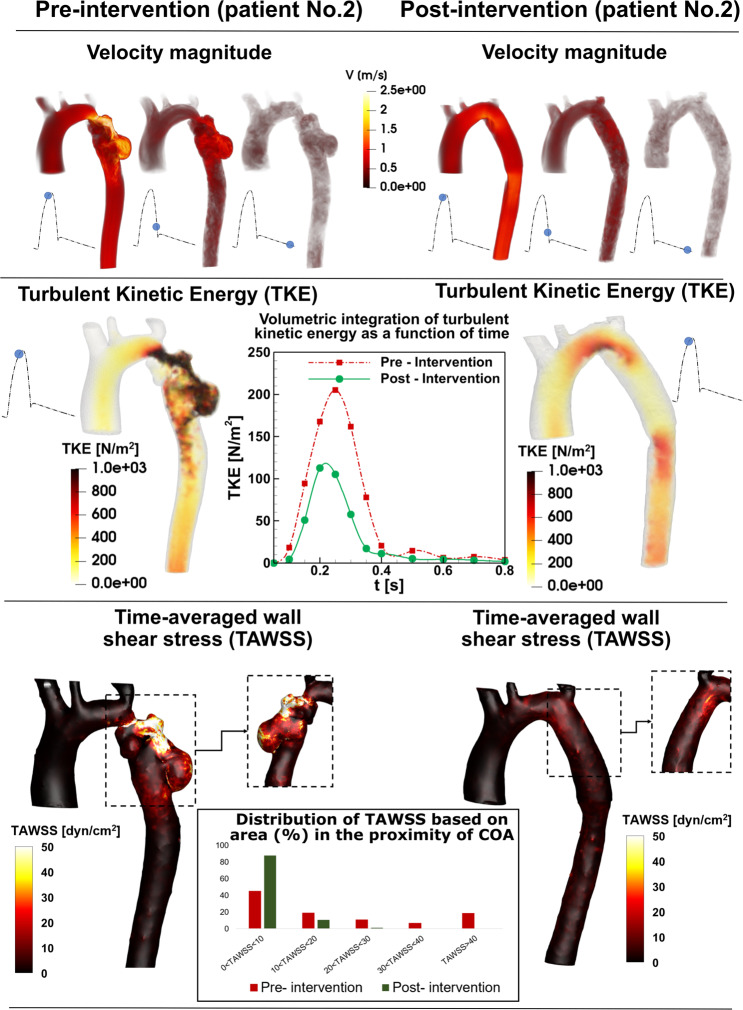
*Flow modeling in Patient No. 2*. Computed velocity magnitude, turbulent kinetic energy and time-averaged wall shear stress using the computational framework (based on LPM and LBM) in pre and post intervention status in Patient No. 2. Patient No. 2 underwent intravascular stent intervention to correct the coarctation which was coexisted with a major aneurysm downstream of the coarctation. Post intervention, the stent was successfully deployed without residual stenosis. Angiography and pressure measurement confirmed stent expansion with no extravasation, contrast staining or hemodynamic instability. There was no evidence of aneurysm and the patient tolerated the procedure well without complication.

**Figure 9:**
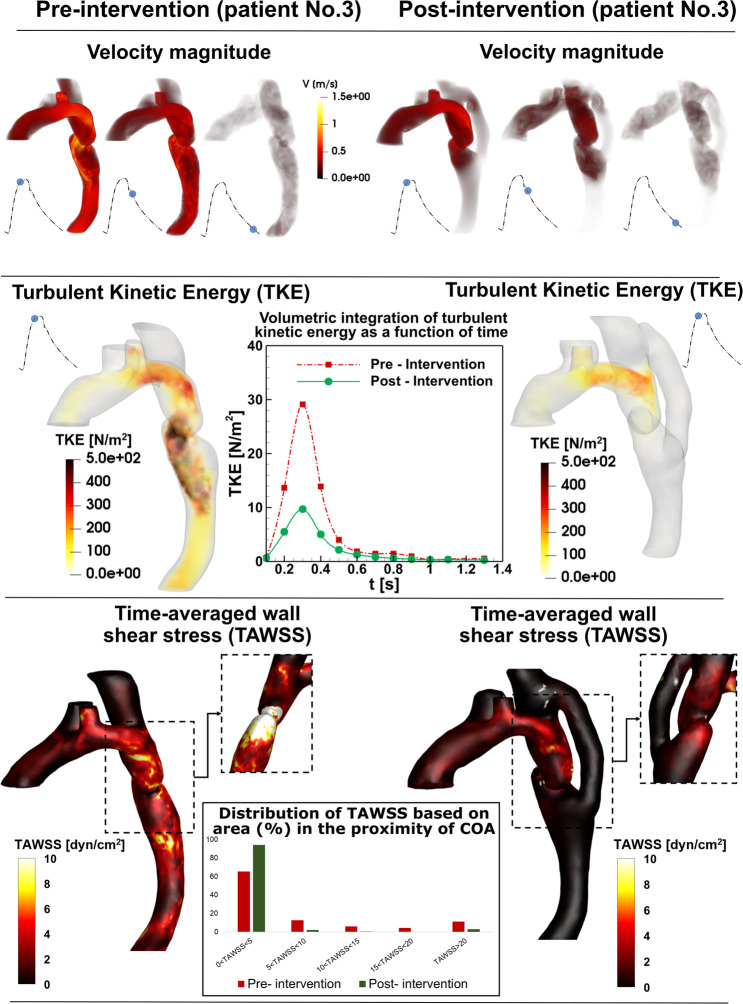
*Flow modeling in Patient No. 3*. Computed velocity magnitude, turbulent kinetic energy and time-averaged wall shear stress using the computational framework (based on LPM and LBM) in pre and post intervention status in Patient No. 3. Patient No. 3 underwent bypass grafting intervention to correct the coarctation. The patient tolerated the procedure well without complication and the intervention was performed successfully.

The presence of the COA modified substantially the flow dynamics and vortical structure in the aorta. As the flow exited the COA, the fluid cannot immediately change direction and followed the steep curvature to reattach to the descending aorta wall (Figs. 7 to 9). Indeed, the disturbed flow resulting from COA detached from the walls and developed into a high-speed and eccentric jet with maximal velocities of: 2.45, 7.5 and 1.47 m/s, creating transitional to turbulent flow downstream of COA with maximum Reynolds numbers of 8400, 13846 and 6203 in Patients No. 1 to 3, respectively (Figs. 7 to 9). Following intervention, the flow pattern was smooth with a relatively low magnitude and more attached to the wall with maximum velocities of: 2.84, 1.4 and 1.05 m/s and maximum Reynolds numbers of 9737, 5908 and 4431 in Patients No. 1 to 3, respectively (Figs. 7 and 9). In patient No. 1, post intervention, the stent was deployed with mild residual stenosis due to malapposition of the stent proximal to the COA (Fig. 7). This could partly explain why the flow pattern was not improved substantially by intervention.

In order to investigate the onset of instability and the intensity of fluctuations in the fluid flow environment, we specifically elected the turbulent kinetic energy (TKE), which is derived using fluctuating components of the velocities and is a sum of the normal fluctuating stresses^52^. Both TKE contours and volumetric integration of TKE during cardiac cycle were reduced in Patients No. 2 and 3 (Figs. 8 and 9) while they were not improved in Patient No. 1 (Fig. 7) by intervention. Note that volumetric integration of TKE during the cardiac cycle can quantify the level of fluctuations in the flow field through the aorta. In Patients No. 2 & 3 (pre intervention), the strong jet due to the presence of the COA generated high fluctuations in the flow field as evident from the high magnitudes of TKE. This adverse condition was alleviated by intervention (Patient No. 2: peak TKE in pre intervention = 1150 N/m^2^, peak TKE in post intervention = 820 N/m^2^, 29% decrease; Patient No. 3: peak TKE in pre intervention = 440 N/m^2^, peak TKE in post intervention = 290 N/m^2^, 34% decrease). In Patient No. 1, TKE didn’t reduce and didn’t improve by intervention (peak TKE in pre intervention = 890 N/m^2^, peak TKE in post intervention = 920 N/m^2^, 3% increase).

Such flow alterations contributed to elevated wall shear stress mainly at the neck of the COA as well as distal to the COA; the total shear stress exerted on the aorta wall was evaluated using time-averaged wall shear stress (TAWSS). Local perturbation in shear stress exposes endothelial cells to high shear stress which affects vessel distensibility and compliance and potentially lead to vascular diseases^53^. Heterogeneous changes in WSS indices occurred both proximal and distal to the coarctation region prone to atherosclerotic plaque development^54,55^ which may lead to aortic wall complications such as rupture, aneurysm and aortic dissection^56–59^. Similar to TKE, TAWSS was reduced modestly by intervention in Patients No. 2 and 3 (Figs. 8 and 9), moving the flow slightly farther from pure oscillatory to more stable domains (Patient No. 2: peak TAWSS in pre intervention = 197 dyn/cm^2^, peak TAWSS in post intervention = 21 dyn/cm^2^, 89% decrease; Patient No. 3: peak TAWSS in pre intervention = 46 dyn/cm^2^, peak TAWSS in post intervention = 13 dyn/cm^2^, 71% decrease). However, TAWSS was not improved and rose in Patient No. 1 following the intervention: elevated TAWSS is noticed at COA region and downstream of the COA (Fig. 7; peak TAWSS in pre intervention = 31 dyn/cm^2^, peak TAWSS in post intervention = 49 dyn/cm^2^, 58% increase).

In addition to shear stress, the arterial vessel is subjected to another major hemodynamic force, pressure. Presence of COA induces an increase in the pressure drops at the neck of the COA in pre intervention states in all three patients (see Fig. 2 for one example, Patient No. 1). This is very important since wall expansion, compression and collapse are caused by high pressure drops in the COA. Moreover, the pressure drops introduced by the presence of the COA must be compensated by the left ventricle, this in turn can lead to heart failure. Such high-pressure drops were reduced by intervention in all 3 patients, documented by catheter measurements as well as LPM simulations (see Fig. 2 for one example, Patient No. 1).

### LV fluid dynamics (Global hemodynamics)

LV stroke work represents the energy that the ventricle delivers to the blood during ejection and is an effective metric of LV load and clinical state. In Patients No. 2 & 3, LV workload and peak LV pressure were reduced following the intervention: Patient No. 2: LV workload: by 23% & LV peak pressure: by 8%; Patient No. 3: LV workload: by 16% & LV peak pressure: by 13% (Table 3). However, in Patient No. 1, the modest reduction of the pressure drop was not accompanied by reduction in LV function parameters: LV workload and peak LV pressure were increased by 15% and 7.3%, respectively (Table 3). Our results reveal that though pre-intervention COA increases the burden on the left ventricle with augmented flow resistance, post-intervention, the LV load does not improve as introducing a stent reduces the arterial systemic compliance, in fact increasing LV load. Percutaneous stenting of the aorta in Patient No. 1, therefore, had limited efficacy in reducing myocardial stress.

**Table 3:** Heart-function metrics in Patients No. 1 to 3.

Cases		LV workload (J)	LV peak pressure (mmHg)
Patient #1	Pre-intervention	1.086	127.5
	Post-intervention	1.249	136.8
Patient #2	Pre-intervention	1.51	148.4
	Post-intervention	1.16	136
Patient #3	Pre-intervention	1.42	137
	Post-intervention	1.19	119

LV workloads and LV peak pressures resulted from lumped parameter modeling in Patients No. 1 to 3.

## Discussions

Quantification of the complex flow in COA plays an essential role in accurate and early diagnosis which may help the clinician optimize the planned interventions but we still lack proper diagnostic methods for COA in clinics because the fluid-dynamics methods that can be used as engines of new diagnostic tools are not well developed yet. Currently, clinical decisions are largely made based on the anatomy^9^. To augment anatomical information, clinics relies largely on data obtained by cardiac catheterization to evaluate pressure and flow through heart and circulatory system but this is invasive, expensive, and high risk and therefore not practical for diagnosis in routine daily clinical practice or serial follow-up examinations^60,61^. Most importantly, cardiac catheterization only provides access to the blood pressure in very limited regions rather than details of the physiological pulsatile flow and pressures throughout the heart and the circulatory system. Phase-contrast magnetic resonance imaging can provide 3-D velocity field but it has poor temporal resolution^62–64^, is costly, lengthy and not possible for many patients with implanted devices. Doppler echocardiography (DE) is potentially the most versatile tool for hemodynamics diagnosis^65–67^. Although there are some promising 2-D Doppler echocardiography methods^68–71^, 2-D velocity field does not represent 3-D velocity field. On the other hand, existing 3-D Doppler echocardiography techniques suffer from low temporal resolution and there is no 3-D Doppler ultrasound to precisely quantify velocity field. Recent advances in DE velocity measurements are: (1) Echo-PIV is an adaptation of Particle Image Velocimetry (PIV) for computing flow velocity by tracking speckles often enhanced with contrast agents (microbubbles)^69–71^. Echo-PIV is promising but depending on the acquisition frame rate, high velocities can be underestimated^72^, which has implications for diagnosis. In addition, the contrast agent must constantly and homogeneously fill the field to avoid both saturated and dark areas. These may hinder routine clinical application of the method^73^.(2) Colour-Doppler vector flow mapping (VFM) permits calculation of the velocity field without contrast agents through colour DE^68^. Colour DE is fast and routinely used in clinics^73^ but it cannot measure velocity in the direction perpendicular to the beam.

In this study, we developed an innovative fast computational-mechanics and imaging-based framework, using turbulent LBM and LPM, that can eventually, upon further development and validation, function as the main component of new diagnostic methods for complex lesions such as COA. Our proposed framework can investigate and quantify effects of COA on both local and global hemodynamics. The diagnostic information, that the framework can provide, is vitally needed to improve clinical outcomes, to assess patient risk and to plan treatment.

## Limitations

This study was performed on 3 patients with COA in both pre and post intervention states (6 cases). Future studies must consider further validation of the computational framework in a larger population of COA patients. However, our results in this study demonstrate the ability of the framework to track changes in both cardiac, and vascular status before and after intervention. We also observed good agreements between the velocity fields calculated by our proposed framework and the MRI-measured velocity fields (in progress for our other study). These observations made us more confident that the limitation in the number of patients in this study does not affect our conclusions. Moreover, we implemented a novel approach to improve the accuracy of computing WSS in LBM models^45^. However, there is room for improving WSS calculations in LBM to be more comparable to those calculated using finite-volume based methods which we will consider in future studies.

## Data Availability

All data, code and algorithms used for this study are available from the author upon request.
